# IgG4‑related disease with endobronchial nodules: A case report and literature review

**DOI:** 10.3892/mi.2025.227

**Published:** 2025-03-18

**Authors:** Cheuk Cheung Derek Leung, Yu Hong Chan, Man Ying Ho, Yiu Cheong Yeung

**Affiliations:** Department of Medicine and Geriatrics, Princess Margaret Hospital, Hong Kong, SAR, P.R. China

**Keywords:** IgG4-related disease, IgG4-related lung disease, endobronchial nodules, storiform fibrosis

## Abstract

The present case report describes the case of a rare presentation of IgG4-related lung disease (IgG4-RLD) with endobronchial nodules and systemic involvement. An 84-year-old male presented with unexplained weight loss and enlarged submandibular lymph nodes. Diagnostic examinations revealed elevated serum IgG4 levels, along with findings of endobronchial nodules during bronchoscopy. Biopsies of endobronchial nodules revealed benign bronchial mucosa with fibrosis and inflammatory infiltration; however, he was not diagnosed with IgG4-RLD. A histological examination of the left salivary gland and submandibular lymph nodes confirmed the diagnosis of IgG4-related disease, revealing storiform fibrosis and abundant IgG4-positive plasma cells. The patient was treated with prednisolone, resulting in a reduction in submandibular swelling and decreased serum IgG4 levels. This case emphasizes the importance of considering IgG4-RLD as a potential diagnosis in patients presenting with endobronchial nodules and systemic disease. Accurate sampling methods, such as surgical lung biopsy or transbronchial cryobiopsies, are crucial for definitive diagnosis. Increased awareness among physicians can lead to timely recognition and appropriate management of this rare condition.

## Introduction

IgG4-related disease (IgG4-RD) is a rare, immune mediated-fibroinflammatory condition that primarily affects patients of middle to advanced age and is more common among males than females (1). IgG4-RD was initially described in 1951 as chronic autoimmune pancreatitis (2,3). In 2003, upon the discovery of extra-pancreatic lesions featuring IgG4-positive plasmacytic infiltration in patients with autoimmune pancreatitis, IgG4-RD was proposed as a novel clinicopathological entity (4). In 2019, IgG4-RD was formally defined by a classification criteria (5). IgG4-RD has been described in virtually every organ and is characterized histologically by the infiltration of immunoglobulin G4 (IgG4)-bearing lymphoplasmacytic cells (6). Respiratory manifestations, or IgG4-related lung disease (IgG4-RLD), encompass various features, such as the presence of solid nodules in the lung parenchyma, the thickening of bronchovascular bundles and interlobular septa, and rounded ground glass opacities (7). The present study describes an uncommon case of IgG4-RLD with endobronchial nodules, as well as systemic involvement.

## Case report

An 84-year-old retired electrician with a previous medical history of ischemic heart disease, hypertension and gout was referred to the general medical outpatient clinic of Princess Margaret Hospital, Hong Kong, SAR, China due to a 6-month history of unquantified weight loss. He had no fever, night sweats or any respiratory symptoms. A physical examination of his abdomen, cardiovascular and respiratory systems did not reveal any notable findings, apart from multiple enlarged submandibular lymph nodes. The patient recalled a diagnosis of IgG4-RD more than a decade ago with no treatment offered and claimed there was no marked progression of submandibular swelling over the years.

**Figure 1:**
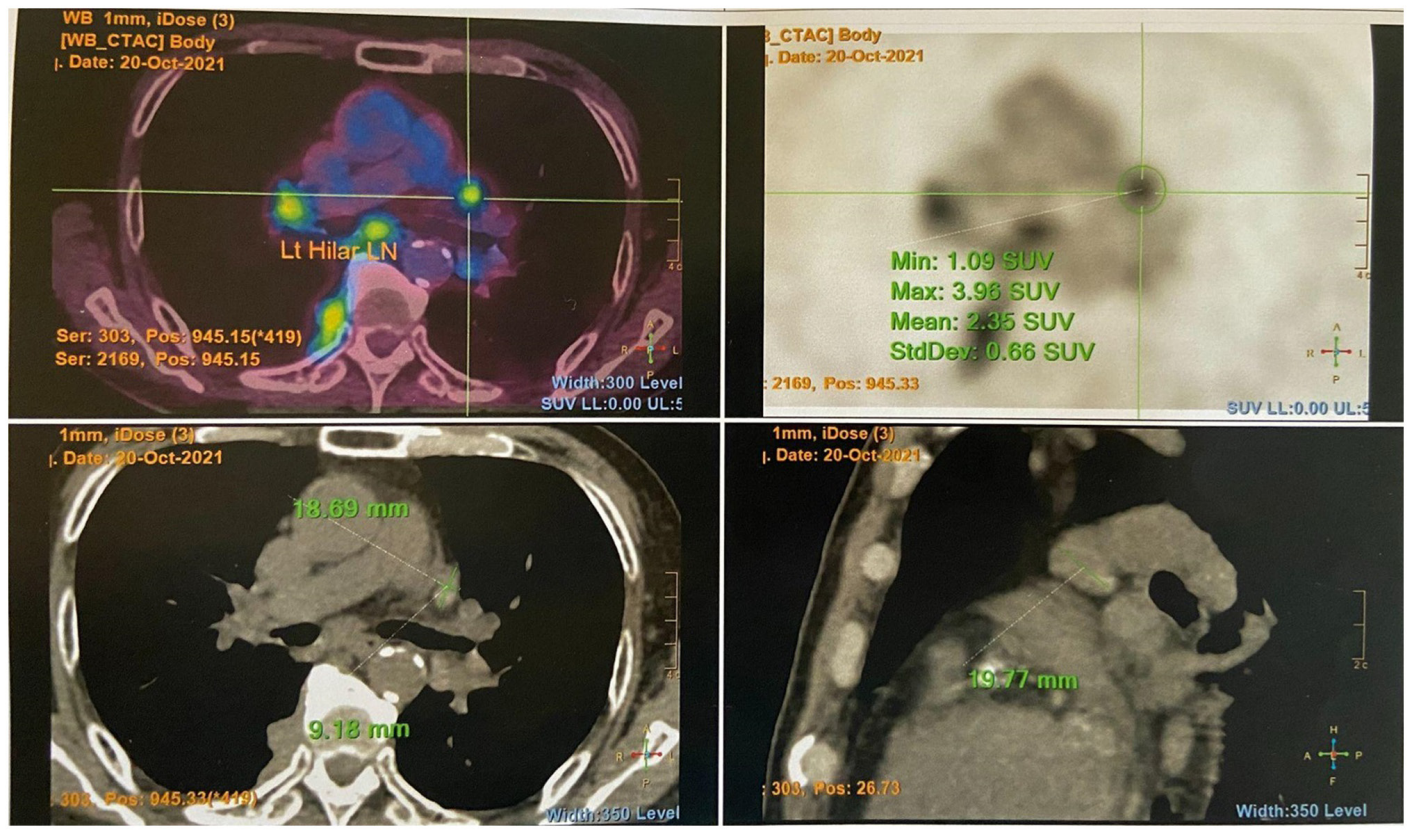
PET/CT image illustrating multiple FDG-avid enlarged mediastinal and hilar lymph nodes. The top left image is a transverse cut fusion (PET/CT) image. The top right image is a transverse cut PET image. The bottom left image is a plain CT transverse cut image. The bottom right image is a plain CT sagittal cut image. PET/CT, positron emission tomography-computed tomography.

**Figure 2:**
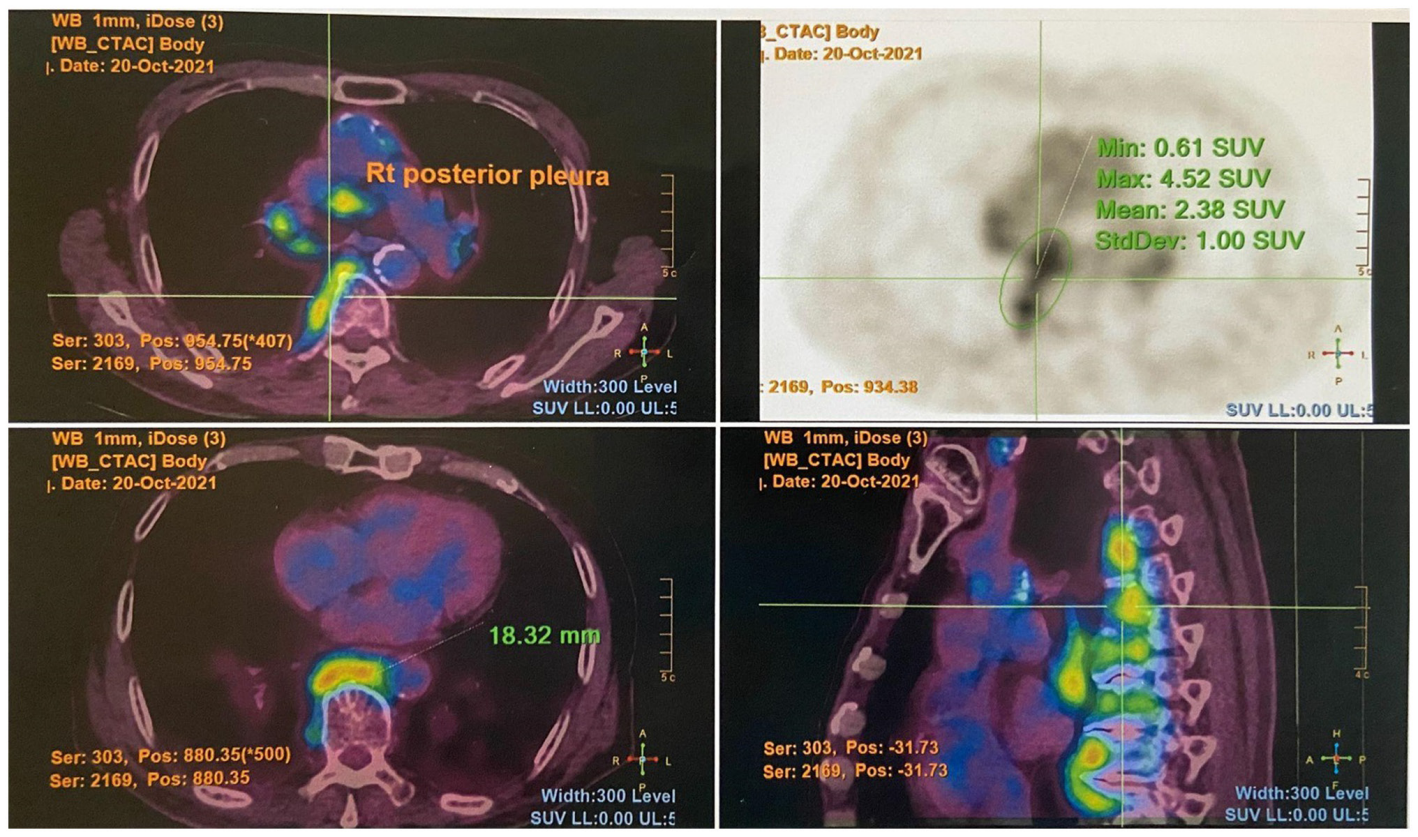
PET/CT image illustrating an FDG-avid nodular thickening of the right posterior pleura at the T9/10 level. The top left image is a transverse cut fusion (PET/CT) image at the mid-thoracic level. The top right image is a transverse cut PET image. The bottom left image is a transverse cut fusion (PET/CT) image at the lower thoracic level. The bottom right image is a sagittal cut fusion (PET/CT) image. PET/CT, positron emission tomography-computed tomography.

The levels of tumor markers, including alpha fetal protein (3 µg/l), prostate-specific antigen (<0.03 µg/l) and carcinoembryonic antigen (1.9 µg/l) were withiun normal range. He had an increased eosinophil count (1.9x10^9^/l; reference range, 0.0-0.5x10^9^/l) and an erythrocyte sedimentation rate of 119 mm/h (reference range, <32 mm/h). Autoimmune markers including antinuclear, antineutrophil cytoplasmic, Sjögren syndrome-related antigen A/Ro, Sjögren syndrome-related antigen B/La, double-stranded DNA, ribonucleoprotein and Smith antibodies were all negative. His serum IgG4 count was increased (2,269 mg/dl; reference range, 9-146 mg/dl). A positron emission tomography-computed tomography scan revealed multiple fludeoxyglucose F18 (FDG)-avid enlarged mediastinal, hilar, (Fig. 1) and submandibular lymph nodes, as well as an FDG-avid right parotid gland nodule. An FDG-avid nodular thickening of the right posterior pleura was also noted at the T9/10 level (Fig. 2).

**Figure 3:**
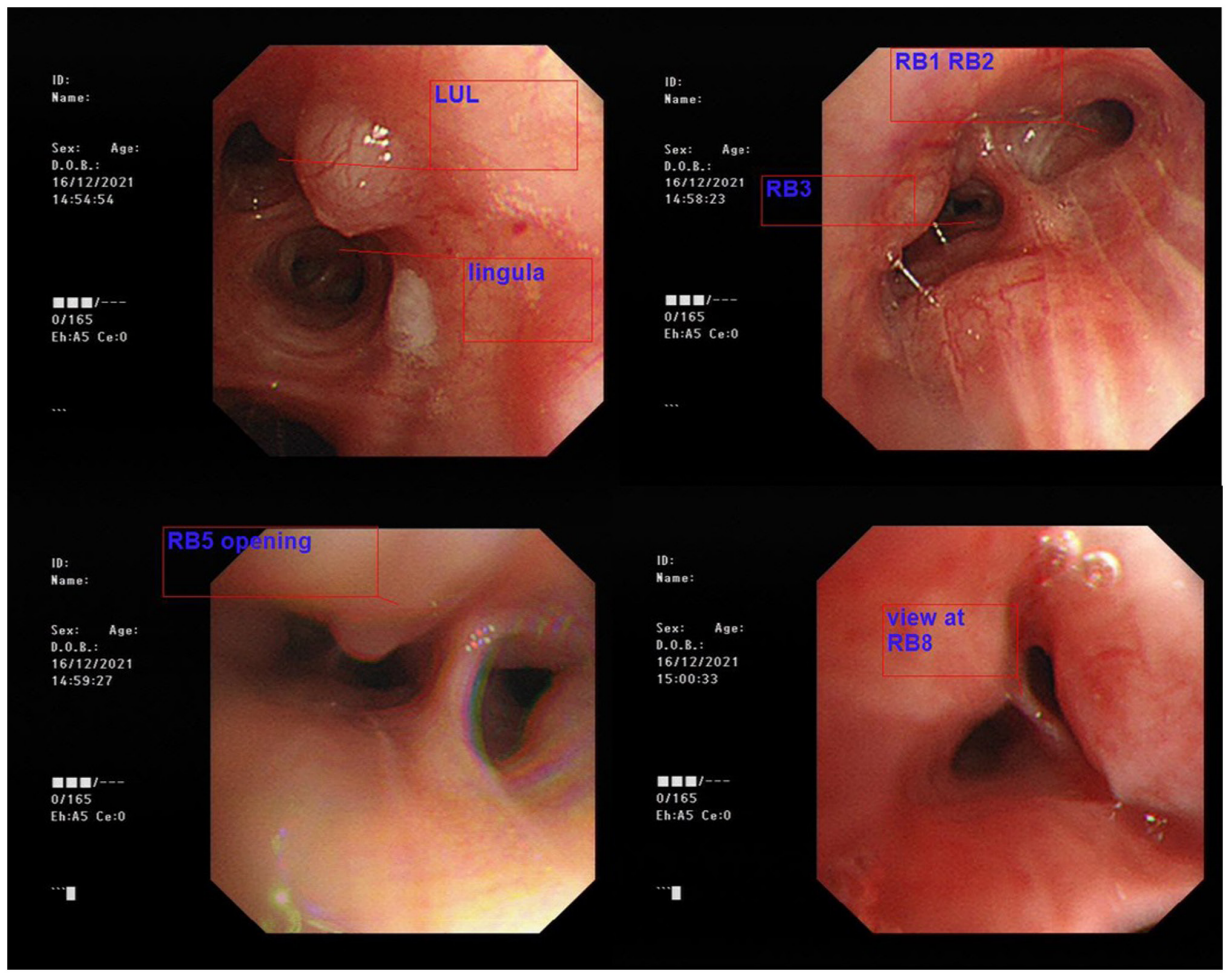
Flexible bronchoscopy image of diffuse submucosal nodular swelling in both bronchi. LUL, left upper lobe; RB1, right upper lobe apical bronchus; RB2, right upper lobe posterior bronchus; RB3, right upper lobe anterior bronchus; RB5, right middle lobe medial bronchus; RB8, right lower lobe anterior basal bronchus.

Flexible bronchoscopy incidentally identified diffuse submucosal nodular swelling in both bronchi, featuring a smooth surface continuous with the respiratory tract mucosa (Fig. 3). Multiple endobronchial biopsies of nodules were taken at the right upper lobe anterior bronchus (RB3) and right lower lobe anterior basal bronchus (RB8). An endobronchial ultrasound (EBUS) identified enlarged station 4R and station 10 lymph nodes, with transbronchial needle aspiration (TBNA) performed over the station 4R lymph node. The cytology of the EBUS-TBNA sample only revealed tiny fragments of crushed lymphoid tissue with no granuloma or malignant cells. The histological analysis of multiple endobronchial biopsies revealed benign bronchial mucosa with stromal fibrosis and containing patchy inflammatory infiltration with marked crushing artefact (data not shown).

**Figure 4:**
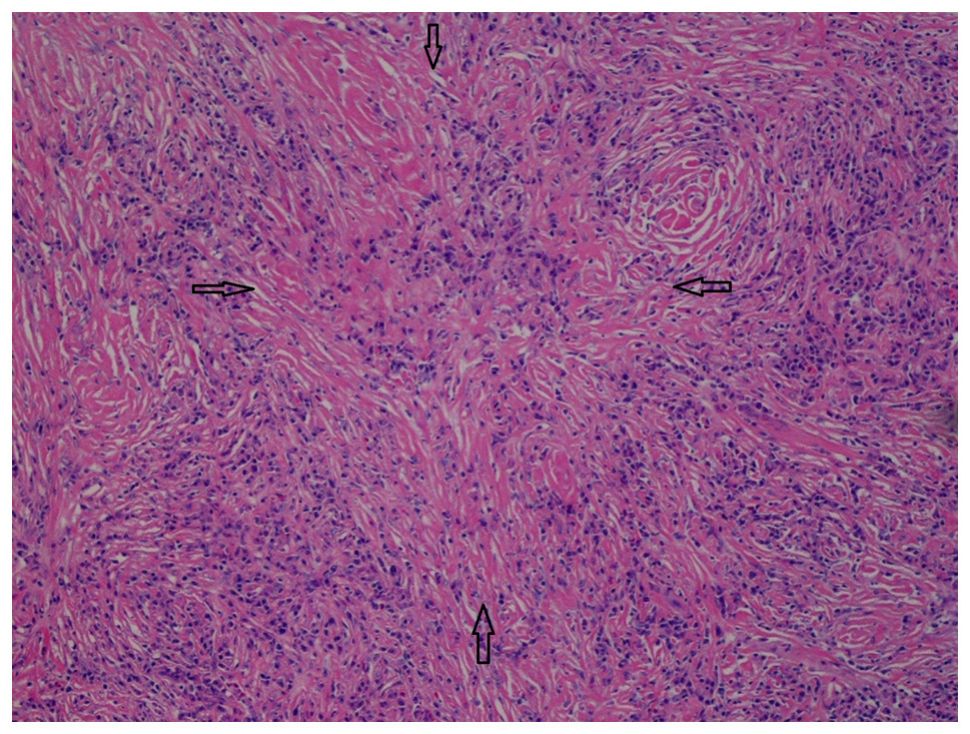
Histopathological slide (hematoxylin and eosin staining, x100 magnification) of the left salivary glandular tissue showed storiform fibrosis, a feature of IgG4-RD.

In view of the diagnostic uncertainty, a left-sided submandibular sialoadenectomy with lymph node excision was performed by the surgeons. A histological examination of the left salivary glandular tissue (hematoxylin and eosin staining; performed by the Histopathology Laboratory of Yan Chai Hospital, Hong Kong, SAR, China; x100 magnification) revealed evidence of chronic sclerosing sialadenitis (Fig. 4). The salivary gland lobules were mostly replaced by storiform fibrosis, reactive lymphoid hyperplasia and lymphoplasmacytic infiltration. Plasma cells were abundant. Obliterative phlebitis was not observed. There was no evidence of malignancy. Immunohistochemistry performed by the Histopathology Laboratory of Yan Chai Hospital revealed a high number of IgG4 plasma cells >100/HPF in the most affected area and the IgG4:IgG plasma cell ratio was >40% (the images for this were not available). All these features were in-keeping with IgG4-RD. Similar features were also observed in the sampled submandibular lymph node.

After the diagnosis of IgG4-RD was made, the patient was treated with prednisolone 10 mg daily (body weight, 63.5 kg) with a gradual tapering regimen. There was a marked reduction in submandibular swelling and his weight remained static. At the latest follow-up, the serum IgG4 level decreased from 2,269 to 314 mg/dl after the 10 weeks of treatment.

## Discussion

Various international diagnostic criteria for IgG4-RD are available, including the 2019 American College of Rheumatology/European League Against Rheumatism (ACR/EULAR) Classification Criteria for IgG4-Related Disease, and the 2020 Japan College of Rheumatology's revised comprehensive diagnostic (RCD) criteria for IgG4-RD (5,8). The RCD criteria is the most updated criteria, requiring fulfilment of clinical and radiological, serological and pathological features (Table I). The case described herein fulfils the RCD criteria of definite IgG4-RD.

The diagnostic criteria used in IgG4-RLD is the same as IgG4-RD. Due to its rarity, the exact prevalence of IgG4-RLD is not known; however, studies have reported lung involvement in 2.4 to 27.1% of IgG4-RD cases (9). Patients with IgG4-RLD typically exhibit minimal respiratory symptoms. Its diagnosis is often incidental, discovered during investigations for extrathoracic lesions or as an unexpected finding of abnormal lung shadows (10). Characteristic features of IgG4-RLD on computed tomography scans include hilar lymphadenopathies and the presence of soft tissue masses in the paravertebral region (11). Histopathological findings of IgG4-RD are dense lymphoplasmacytic infiltrate, storiform fibrosis, obliterative phlebitis, and increased numbers of IgG4-positive plasma cells or an increased IgG4:IgG ratio in tissue (6).

Numerous cases of systemic IgG4-RD associated with tracheobronchial edema, or capillary dilatation have been reported, in addition to IgG4-RLD cases featuring isolated tracheobronchial involvement, manifesting as mass-like lesions (7,12,13). IgG4-RLD presents as multiple endobronchial nodules; however, it is extremely rare. To the best of our knowledge, only two previous articles to date have reported IgG4-RLD with multiple endobronchial nodules (7,14). The first case reported by Wang *et al* (7) involved a 52-year-old male patient who presented with wheezing accompanied by jelly-like sputum. His spirometry results were normal, and a computed tomography scan of the thorax revealed left lower lung infiltrate without obvious bronchial nodules. A bronchoscopy revealed multiple white nodular protuberances in the trachea and bronchus, with mucosal edema and hyperemia. He was diagnosed with IgG4-RLD histologically through standard forceps biopsy and was treated with prednisolone and azathioprine. There was no evidence of involvement of other organs due to IgG4-RD (7). The second case reported by Torii *et al* (14) involved a 74-year-old female patient with known IgG4-RD affecting the parotid gland and eyelids. She had previously been treated conservatively, with spontaneous resolution of her symptoms. After 3 years, she presented with a recurrence of swelling in the parotid gland and eyelids, which this time, was accompanied by a cough, an obstructive pattern on spirometry, and a computed tomography scan of the thorax revealed extensive multiple nodules in the trachea and bronchi, without any masses or ground-glass nodules in the lung parenchyma. A bronchoscopy revealed multiple nodules with a smooth surface, continuous with the respiratory tract mucosa. A diagnosis was made by an endobronchial cryobiopsy and she was treated with prednisolone (14). Both these aforementioned cases had a resolution of endobronchial nodules demonstrated by repeat bronchoscopies. In contrast to the two cases mentioned above, the patient in the present study had no respiratory symptoms. Spirometry was not performed as a result, and his computed tomography scan of the thorax did not reveal obvious bronchial nodules. Similar to the case reported by Torii *et al* (14), the patient in the present study exhibited systemic involvement of IgG4-RD with endobronchial nodules that had a smooth surface continuous with the respiratory tract mucosa.

The limitations of the present study include the lack of a follow-up bronchoscopy to prove the resolution of endobronchial nodules following the use of steroids, and no pathological confirmation of IgG4 plasma cells in the endobronchial nodules. However, it was deemed that there were no alternative explanations of the endobronchial nodule.

A histological analysis is vital in diagnosing IgG4-RLD, as the serum IgG4 level may be raised in several other conditions, including multicentric Castleman's disease, repeated infections, autoimmune diseases, cancer, primary immunodeficiencies and systemic vasculitis (15,16). In the case described herein, biopsies of endobronchial nodules had crushing artefacts and were not diagnostic of IgG4-RLD. EBUS-TBNA also failed to reveal specific features. The diagnosis was only made following a histological examination of the left salivary glandular tissue which revealed characteristic IgG4-RD changes. A previous study demonstrated that histological findings from transbronchial lung biopsies supported the diagnosis of IgG4-RLD in 47% of the cases only (17). Apart from the obvious selection of surgical lung biopsy when diagnostic uncertainty is met, transbronchial cryobiopsies have been reported to successfully diagnose IgG4-RLD (12,14).

The treatment for IgG4-RLD is the same as that for IgG4-RD. Glucocorticoids prove to be a potent remedy for IgG4-RD. However, instances of disease resurgence often occur either during or after the dose reduction of glucocorticoids, and these drugs rarely result in sustained periods of remission without further treatment (18). Rituximab, a monoclonal anti-CD20 antibody, was shown to be effective as both induction therapy and treatment of relapses in IgG4-RD in a retrospective nationwide study in France (19). Inebilizumab, a monoclonal anti-CD19 antibody, demonstrated reduction in the risk of IgG4-RD flare up and increased the likelihood of complete remission without flares at 1 year (20). For the patient in the present study, long-term follow-up is necessary to monitor for disease recurrence following the discontinuation of prednisolone. Biological agents should be considered if remission is not achieved.

In conclusion, the present study reported a rare presentation of IgG4-RLD with multiple endobronchial nodules and systemic involvements, found incidentally during flexible bronchoscopy. A histopathological examination of the endobronchial biopsy was inconclusive and diagnosis was made after surgically sampling the left salivary gland and submandibular lymph nodes. This case serves the purpose of reminding physicians to consider IgG4-RLD as a differential diagnosis when endobronchial nodules are observed with or without systemic disease, and appropriate sampling methods should be selected to increase its diagnostic yield.

## Figures and Tables

**Table I:** The 2020 Revised Comprehensive Diagnostic (RCD) criteria (8) for IgG4-RD.

1. Clinical and radiological features
- One or more organs show diffuse or localized swelling or a mass or nodule characteristic of IgG4-RD. In single-organ involvement, lymph node swelling is omitted.
2. Serological diagnosis
- Serum IgG4 levels >135 mg/dl.
3. Pathological diagnosis
- Positivity for two of the following three criteria:
- Dense lymphocyte and plasma cell infiltration with fibrosis.
- Ratio of IgG4-positive plasma cells /IgG-positive cells greater than 40% and the number of IgG4-positive plasma cells greater than 10 per high powered field
- Typical tissue fibrosis, particularly storiform fibrosis, or obliterative phlebitis
Diagnosis:
Definite: 1 + 2 + 3
Probable: 1 + 3
Possible: 1 + 2

IgG4-RD, IgG4-related disease.

## Data Availability

The data generated in the present study may be requested from the corresponding author.
